# Quantification of Antibiotic Concentration at the Site of Infection: Analytical Challenges and Current Practices

**DOI:** 10.1097/FTD.0000000000001501

**Published:** 2026-08-18

**Authors:** Soma Bahmany, Amanda J. Holst, Wichor M. Bramer, Rawi Ramautar, Robert. B. Flint, Birgit C. P. Koch

**Affiliations:** * Department of Hospital Pharmacy, Erasmus MC, University Medical Center, Rotterdam, the Netherlands;; † Medical Library, Erasmus MC-Erasmus University Medical Center, Rotterdam, the Netherlands.; ‡ Leiden Academic Center for Drug Research, Leiden University, Leiden, the Netherlands; and; § Department of Pediatrics, Division of Neonatal and Pediatric Intensive Care, Erasmus MC Sophia Children's Hospital, Rotterdam, the Netherlands;; ¶ CATOR, Center for Antimicrobial Treatment Optimization, Rotterdam, the Netherlands;

**Keywords:** anti-infective agents, microdialysis, target-site quantification, tissue biopsy, challenges

## Abstract

**Background:**

Quantifying antibiotic concentrations at infection sites is challenging because of invasive sampling and complex matrices. Despite limited knowledge, target-site data are crucial for optimizing dosing, minimizing resistance, ensuring safer strategies, and reducing toxicity risks through adequate target attainment.

**Methods:**

This review aims to provide an overview of the most commonly reported challenges associated with quantifying target-site concentrations of antibiotics. Antibiotics were selected as the focus because of their use in hospital and outpatient settings. An extensive literature search was conducted using Embase.com. After title and abstract screening of 828 articles, 398 articles were included for full-text screening, and 72 were included based on relevance to analytical challenges in target-site quantification. Reported challenges were categorized in subgroups: sampling difficulties, sample heterogeneity, unavailability of free drug concentrations, drug complexities, and others.

**Results:**

Direct measurement of antibiotic concentrations at infection sites is constrained by invasive procedures and complex analytical methods. Key challenges include sampling heterogeneity, difficulty in obtaining samples, and the lack of free drug concentration data. Several opportunities have been proposed to address these challenges.

**Conclusions:**

We recommend updating international validation guidelines to include standardized tissue sampling protocols, encouraging the commercial sectors to develop reference standards and blank materials for alternative matrices, and promoting the use of minimally invasive techniques, such as microneedle biosensors, to improve the accuracy and safety of target-site quantification. These advances offer promising avenues to enhance antimicrobial therapy optimization despite current.

## Abstract 2

Supplemental Digital Content is Available in the Text.

## INTRODUCTION

Adequate antibiotic exposure at the site of infection is crucial for the successful treatment of bacterial infections. Inadequate local antimicrobial concentrations are associated with increased antibiotic resistance, treatment failure, and prolonged infection duration.^1^ Conversely, excessively high concentrations may result in severe adverse effects and potentially irreversible damage.^2^ Currently, therapeutic drug monitoring (TDM) primarily relies on plasma concentrations, which are often used as surrogate markers. However, plasma concentrations may not accurately reflect antibiotic exposure at the actual site of infection, potentially leading to less effective treatment decisions. Therefore, direct measurement of antibiotic concentrations at the infection site is crucial to accurately assess true drug exposure and provide essential insights for optimizing antibiotic therapy.^3^

Despite the importance of precise dosing, significant knowledge gaps remain regarding antibiotic concentrations at the site of infection. These gaps are primarily because of the invasive nature of sampling procedures and the limited availability of suitable analytical methods. Although several studies have proposed techniques for quantifying target-site concentrations, the complexity of biological matrices at infection sites presents considerable analytical challenges.

The aim of this review is to provide a comprehensive overview of the challenges associated with quantifying antibiotic concentrations at the site of infection. As an academic research center specializing in antibiotic therapies, our focus is on understanding drug exposure in these critical locations. This emphasis is driven by the widespread use of antibiotics in hospital and outpatient settings. Achieving target attainment is crucial for maximizing therapeutic efficacy, minimizing antimicrobial resistance, enabling safer dosing strategies, and reducing the risk of toxicity. In the following sections, we discuss the pharmacological principles underlying antibiotic therapy, review current analytical techniques for measuring target-site concentrations, discuss sampling methodologies for obtaining infection-site matrices, and propose potential solutions to these challenges, as outlined in Section 5.

### Antibiotics

Antibiotics are a class of medications designed to inhibit bacterial growth or directly kill bacteria. They play a crucial role in the treatment and management of infections and in preventing infections during surgeries, cancer chemotherapy, and organ transplantation.^4^ The introduction of antibiotics has significantly reduced the mortality rate associated with bacterial infections.^5^ However, the misuse and overuse of antibiotics contribute to the emergence of antibiotic resistance, in which bacteria adapt and become resistant to previously effective treatments.^6^

Antibiotics are typically classified based on their chemical structures, mechanisms of action, and spectrum of activity. In this review, we categorize antibiotics into major groups, including beta-lactams, aminoglycosides, tetracyclines, macrolides, fluoroquinolones, and glycopeptides, and those that do not fall into these categories are classified as “Others.”

### Analytical Methods

In clinical practice, highly accurate, selective, and sensitive analytical techniques are required for the quantification of antibiotic concentrations in biological matrices. Two commonly used techniques in clinical laboratories are immunoassays and chromatographic methods. Chromatographic methods are often coupled with ultraviolet absorbance detection or mass spectrometry. Immunoassays are analytical techniques that rely on the binding between antigens and antibodies to detect and quantify a variety of biomolecules, including drugs. These immunoassays are widely used in clinical settings because of their rapid turnaround time and minimal sample preparation requirements. However, their main limitations include susceptibility to cross-reactivity and matrix effects, which can undermine accuracy and reliability.^7^

In contrast, chromatographic techniques, particularly when combined with advanced or mass spectrometry detection, offer greater specificity, sensitivity, and accuracy than immunoassays. However, the main drawbacks of chromatographic techniques are their operational complexity, higher equipment costs, the need for sample cleanup, and longer turnaround times.

### Methods for Assessment of Tissue Pharmacokinetics

To accurately quantify target-site antibiotic concentrations, samples must be obtained directly from the site of infection. This requires more complex sampling methods than regular blood sampling methods. Two commonly used sampling techniques are tissue biopsies and microdialysis (MD).

A tissue biopsy involves the removal of a small tissue sample from the body. Various biopsy methods can be used, with needle and surgical biopsies being the most common. In a needle biopsy, a needle is inserted into the infection site to extract tissue or fluid samples from muscles, bones, or other organs. Surgical biopsies involve the collection of tissue samples during surgery.

Microdialysis is another widely used sampling technique for quantifying free target-site concentrations in interstitial space fluid. The principle of MD relies on the diffusion of molecules between 2 fluid compartments separated by a semipermeable membrane within a probe. The probe is implanted into the infected tissue of interest, and a perfusion fluid is continuously pumped through this probe at a specific flow rate. The composition of this perfusion fluid mimics the ionic composition of the interstitial fluid of the body. Because of the difference in concentrations between the perfusion fluid and the tissue interstitial fluid, small molecules, including antibiotics, diffuse across the semipermeable membrane into the perfusion fluid. The collected fluid, known as dialysate, contains the recovered antibiotic and is subsequently analyzed.^8,9^

### Free Drug Concentrations

Antibiotics bound to proteins are generally considered inactive because they cannot penetrate bacterial cell membranes or effectively bind to their targets. Consequently, the pharmacological activity of antibiotics is primarily attributed to their unbound (free) fractions. Therefore, quantifying free drug concentrations is crucial, as these represent the active component responsible for therapeutic effects.^10^ Free drug concentrations are typically measured using techniques such as ultrafiltration, equilibrium dialysis, and MD.

Ultrafiltration involves passing a sample through a filter with a specific molecular weight cut-off to separate bound and unbound drug fractions. This method is faster than equilibrium dialysis and is commonly used in clinical settings.^11^ In equilibrium dialysis, the drug is separated into 2 compartments by a semipermeable membrane, allowing it to equilibrate between the 2 sides. One compartment contains the sample, whereas the other contains a buffer solution. The concentration of the drug in the buffer represents the free drug concentration.^12^ MD is also used to quantify free drug concentrations, as described in Section 1.3.

## LITERATURE SEARCH

### Databases and Searches

A search was conducted on Embase.com in August 2024, with the database last updated in March 2025. The full searches are provided in the Supplementary Materials. We searched for antibiotic agents as major thesaurus terms or terms in the title. Detailed terms can be found in **Supplemental Digital Content 1** (see **Supplementary File 1**, http://links.lww.com/TDM/A960). We combined terms for antibiotics as major thesaurus terms or in the title with terms for tissue drug level or target-site concentration. Over half of the references were with a publication date from before the year 2000. We used a more restricted search for older articles, in which we also focused on tissue drug level or target-site concentration as major thesaurus terms and in the title only. We excluded conference abstracts and animal studies. We limited articles from before 2000 to major thesaurus terms and title words. All results were exported to EndNote, and the Find Full Text option was initiated. For articles where the PDF could not be downloaded automatically, we attempted to manually download the PDF via Google Scholar. A broad range of synonyms for “challenges” was selected to ensure comprehensive coverage during the full-text search. Full-text search was performed using the following terms: challenges, limitations, barriers, facilitators, hurdles, difficulties, and drawbacks. Full-text articles that did not mention any of these terms were not further reviewed.

### Article Screening

Initially, a title and abstract screening was performed after full-text screening. These articles were independently screened by a second reviewer. In cases of disagreements between the 2 reviewers, a third reviewer was asked to screen the questionable articles. Articles were included only if they specifically described challenges related to the analytical quantification of antibiotic concentrations at the target site. The exclusion criteria were articles involving animals, articles not involving antibiotics, or articles addressing challenges related to clinical study designs.

### Data Extraction

Final data processing was performed using Microsoft Excel. All articles included in the review were categorized by group of antibiotics, year of publication, sampling method, analyzed matrix, discussed challenges, and authors' recommendations to correct for the described challenges, if described.

## CHALLENGES DURING THE ANALYTICAL QUANTIFICATION OF TARGET-SITE CONCENTRATIONS: FOCUSED ON ANTIBIOTICS

### Search Outcomes

Initially, the title and abstract of 828 articles were screened. Of these, 430 were found irrelevant, and 398 were selected for full-text screening. Hereafter, full-text screening was performed, which excluded 329 articles. In addition, during the full-text screening of review articles, 3 relevant references related to the described challenges were manually added to the review. Finally, a total of 72 articles were included. Figure 1 presents a flowchart of the inclusion and exclusion criteria.

**FIGURE 1.**
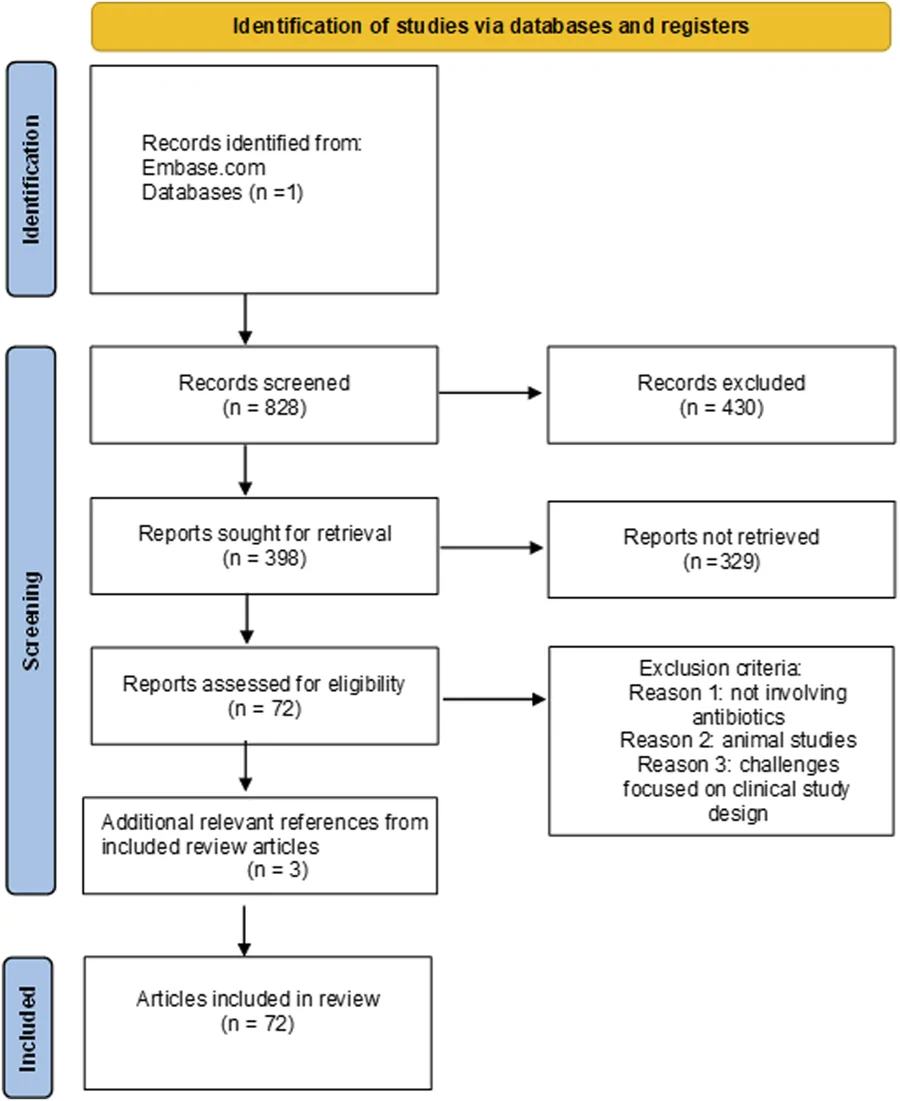
Flowchart of the search strategy and the article screening process.

### Characteristics of Included Studies

Information from the data extraction was collected and filtered in Excel. Study characteristics were filtered by the year of publication, title, antibiotic group, discussed matrices based on the challenge, described matrices for analytical quantification, and potential recommendations provided by the authors.

### Main Challenges of Target-Site Quantifications

In this review, we present an overview of the most commonly reported challenges in the analytical quantification of target-site concentrations of antibiotics. These challenges are described as the main challenges, which were extracted from the literature and categorized into subgroups: sampling difficulties, sample heterogeneity, unavailability of free drug concentrations, drug complexities, and *others*. These main challenges are summarized separately below. A detailed overview of all references, including the corresponding challenges, is presented in **Supplementary Table 1**, http://links.lww.com/TDM/A961.^13–62^ Figure 2 summarizes the main challenges associated with each antibiotic group, and Figure 3 illustrates the impact of these main challenges on TDM practice.

**FIGURE 2.**
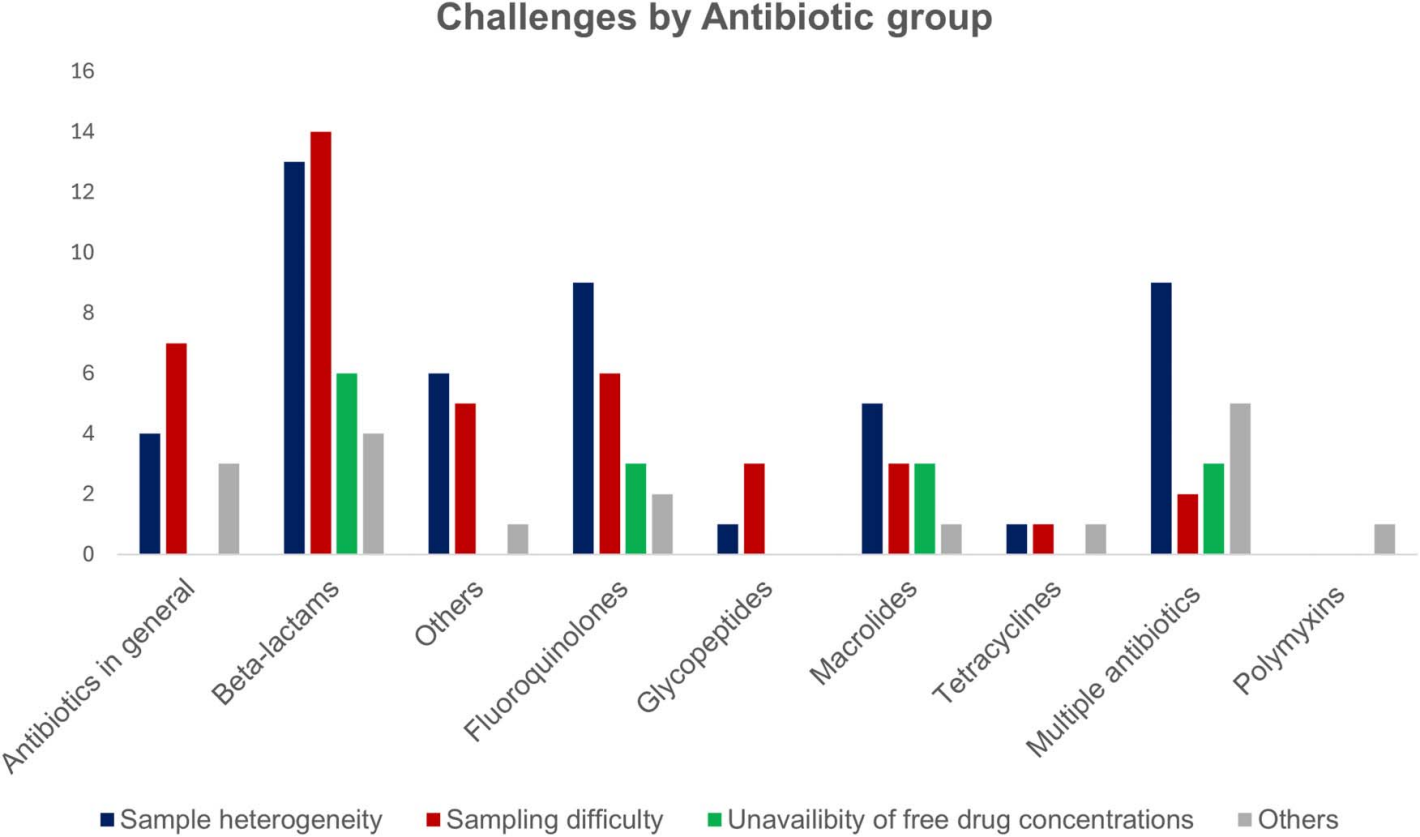
Summary of the main challenges associated with each antibiotic group.

**FIGURE 3.**
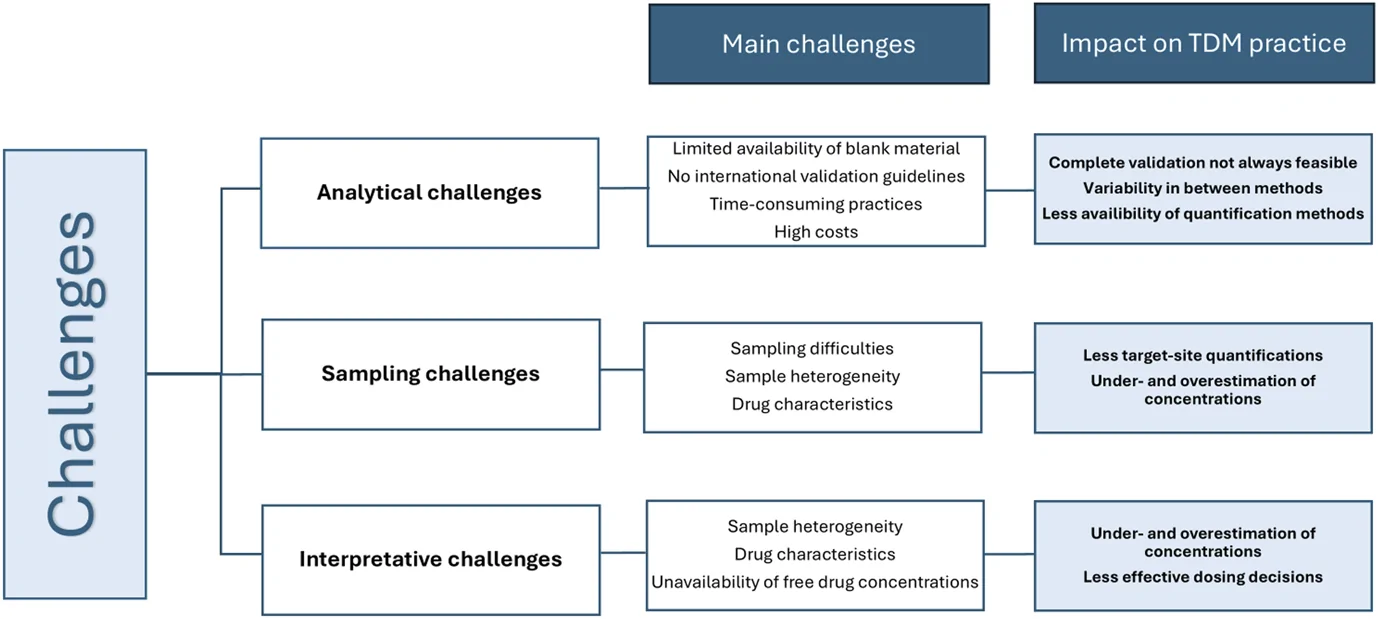
The impact of the main challenges on TDM practice.

#### Sampling Difficulties

As described in the introduction, 2 commonly used sampling methods for target-site concentrations are tissue biopsies and MD. The invasive nature of these sampling methods poses limitations on their clinical applicability, including small sample sizes, restricted sampling time points, and the impracticality of sample reanalysis. Many studies recommend MD for measuring interstitial space fluid concentrations instead of using tissue samples. Nevertheless, MD is also considered invasive, as both tissue biopsies and MD require trained clinical personnel and are typically performed during hospitalization or surgery.

To mitigate the challenges posed by small sample sizes, highly sensitive and selective analytical techniques are recommended.^3,63,64^ The development of more robust pharmacokinetic/pharmacodynamic (PK/PD) models to predict target-site concentrations will help overcome these limitations in the future.^65^

#### Impact of Matrix Composition

##### Sample Heterogeneity

Various types of contamination can occur during the collection of tissue biopsies in clinical practice, making sample heterogeneity a common challenge when quantifying target-site concentrations. Tissue homogenates contain different types of tissue compartments (eg, cellular components, interstitial fluid, cancellous bone, cortical bone, and blood capillaries). For this reason, target-site concentrations of tissues reflect an average concentration of these diverse tissue compartments. Furthermore, tissue samples are mostly contaminated with blood. Despite rinsing the tissues before sample preparation, residual blood may remain, or antibiotics may be washed away during this washing step. As illustrated in Figure 3, these contamination issues may result in underestimation or overestimation of the target-site concentrations, which could lead to misinterpretation of the data. As described by several published studies, corrections can be made for these types of heterogeneities. To correct for blood contamination, hemoglobin concentrations can be measured.^66,67^ Böttcher et al^67^ described a method to determine blood contamination by applying the hexacyanoferrate method from Roche Diagnostics, Mannheim, Germany. Furthermore, positron emission tomography (PET) scanning can be used to discriminate between different tissue compartments.^68–71^

##### Unavailability of Free Drug Concentrations

As described in Section 1.4, free drug concentrations can be obtained using methods such as ultrafiltration, equilibrium dialysis, or MD. These methods are suitable for liquid matrices, such as plasma, cerebrospinal fluid, or interstitial (extracellular) fluids of targeted tissues. However, quantifying free drug concentrations in tissue samples presents significant challenges owing to the complexity of their composition and small sample sizes.^72^ Consequently, the unavailability of free drug concentrations during target-site quantification in tissue samples is frequently cited in the literature as a major challenge, especially for highly protein-bound drugs, which could lead to less effective dosing decisions, as described in Figure 3. Moreover, the extent of protein binding of antibiotics in tissues has not been well characterized.^73^

#### Drug Characteristics

The physicochemical properties of the drug, such as lipophilicity and protein binding, are critical factors to consider when considering the application of MD. For example, antibiotics such as fluoroquinolones and polymyxins may be adsorbed to the probe, tubing, membranes, or other plastic surfaces during MD. This adsorption of drugs to these surfaces may lead to an underestimation of the measured concentrations, as illustrated in Figure 3. Several studies have described this as a main challenge when applying MD for the quantification of free drug concentrations of highly protein-bound antibiotics or lipophilic antibiotics.^8,64,74^ A few studies have recommended performing MD only in cases of hydrophilic antibiotics, such as beta-lactam antibiotics.^8,64^ Matzneller et al^74^ suggested that adsorption may be prevented by adding albumin, beta-cyclodextrin, polysorbate, and organic solvents to the perfusion solutions.

#### Others

In addition to sampling difficulties, sample heterogeneity, the unavailability of free drug concentrations, and drug complexities, several other challenges have been reported.

To quantify antibiotics at the target site, calibration standards and quality control samples should be prepared using blank material containing the matrix of interest. However, complete validation is not always feasible in practice because of the limited availability of blank material.^1,75,76^ In addition, no international guidelines are available for the analytical validation of drugs in tissue samples.^1^ These limitations are associated with the analytical challenges illustrated in Figure 3.

Furthermore, the application of MD in clinical practice also presents certain challenges. Time-consuming calibration and high costs of MD have been described as the main challenges by De La Peña et al and Dhanani et al.^64,77^ In addition, a complete equilibrium between the MD fluid and interstitial fluid is never reached.^78^ De La Peña et al recommended open-flow perfusion as a new technique in which partial equilibration between the interstitial fluid and perfusion fluid occurs.^77^ Other studies have described a correction factor using relative recovery (RR) to account for this error. The RR was calculated using the following equation:

$$\text{RR}=\frac{C_{\text{MD}}\text{fluid}}{C_{\text{tissue}}}$$

The C_MD_ fluid is the concentration in the MD fluid, and the C_tissue_ is the concentration in interstitial fluid. However, this RR correction may also lead to errors.^1^ Another limitation of MD is that the measured concentrations correspond only with extracellular concentrations, as this method cannot measure intracellular concentrations, which may not always truly reflect the concentrations of the infected compartments.^79,80^ Extracellular and intracellular drug concentrations can vary significantly, with intracellular accumulation being crucial for treating infections caused by intracellular pathogens. Antibiotics, such as macrolides and fluoroquinolones, demonstrate significant intracellular concentrations, often surpassing extracellular levels, whereas beta-lactams generally show poor intracellular penetration. Because the infection site exists in different compartments, a combination of different methods is necessary to accurately describe the concentrations at the infected site.^80^ Moreover, during the insertion of the MD catheter, a hole must be introduced in the infected tissue, resulting in dead spaces that may be filled with blood clots and extracellular exudations. It is unknown whether the measured concentration reflects the physiological concentration.^3^

## DISCUSSION

This review discusses several main challenges associated with quantifying target-site concentrations of antibiotics. These main challenges and their impact on TDM practice are illustrated in Figure 3. In this review, we also highlight recommendations from various publications to address some of these challenges. The results show that beta-lactam antibiotics are the most commonly described class in the literature. Furthermore, the data indicate that sample heterogeneity, followed by sampling difficulty, are the most common challenges described. A more detailed overview of the main challenges, including recommendations, is available in **Supplementary Table 2**, http://links.lww.com/TDM/A961. Although corrections for different types of sample heterogeneity are described to improve accuracy, by measuring hemoglobin concentrations or performing a PET scan, they often lead to more time-consuming analyses and higher health care costs. Furthermore, there is a lack of knowledge regarding the application of PET scans for target-site quantification. The clinical implementation of PET imaging for TDM may be limited by its high costs and the restricted availability of PET facilities, as this technology is not available in all hospitals. Considering feasibility, clinicians may need to assess the relevance of these corrections based on their impact on the interpretation of target-site concentrations.

Another challenge that was mainly described, concerns the unavailability of free drug measurements in tissues. MD is frequently recommended for quantifying free drug concentrations, as described in **Supplementary Table 2**, http://links.lww.com/TDM/A961. However, the measured concentrations with MD include only extracellular concentrations, not the total concentration of the infected tissue. Moreover, when considering MD for lipophilic antibiotics, it is important to consider the associated challenges. The literature suggests that MD is more suitable for hydrophilic antibiotics, such as beta-lactam antibiotics.

Equilibrium dialysis may be another possibility; however, it is time-consuming because of the long equilibration times. Furthermore, the percentages of protein binding in tissues have not been well described in the literature. Further studies on protein binding in tissues are needed to calculate the estimated free drug concentrations based on the measured total concentrations in tissues.

Importantly, the challenges described differ in their clinical relevance. Challenges such as the invasiveness of sampling, limited availability of free drug concentrations in tissues, and sample heterogeneity represent major barriers to the clinical implementation of target-site quantification. However, other challenges are mainly methodological or technical and can be more easily solved or adapted.

## EXPERT OPINION AND FUTURE PERSPECTIVES

Based on the information presented in this review, it is clear that there are many opportunities for improvement in overcoming the most commonly described challenges related to target-site concentration quantification. First, clinicians should carefully consider the relevance of target-site concentration measurements based on the severity of the clinical indication, as target-site concentrations may not be needed for all indications but are more important in case of prolonged and severe infections or severe sick patients, particularly in clinical centers specialized in antibiotic therapy. It is essential that clinicians and pharmacists consider the associated challenges and take them into account before making dosing recommendations based on measured target-site concentrations.

An alternative approach for target-site measurements is the use of microneedle biosensors, which are emerging as a novel, minimally invasive technique for real-time monitoring of antibiotic concentrations.^81,82^ Currently, no real-time in vivo biosensor has been approved for clinical use for the TDM of antibiotics; most are still in the proof-of-concept stage.^82^ Standardized validation and calibration protocols and regulatory approval are essential to ensure accuracy, reproducibility, and clinical reliability for the implementation of biosensors in TDM. Nevertheless, the current literature addressing these aspects is relatively scarce.

Furthermore, with the increasing number of methods for target-site quantification, international validation guidelines must also be updated and expanded. These guidelines should outline specific validation parameters and provide more standardized guidelines for the sample preparation of tissue samples (eg, rinsing and pulverization steps) to reduce variations between different methods and minimize sample contamination.

In addition, although quantification methods have been developed for alternative matrices, there is still a lack of available external reference samples for these matrices, such as various types of tissues and body fluids. Moreover, the availability of blank material in these alternative matrices should be improved. To address this gap, the commercial sector should invest more in the development of these resources. Currently, reference calibration standards and quality control samples are primarily available for more common matrices, such as whole blood and plasma. The availability of external reference samples would help better understand the potential deviations caused by various challenges.

Finally, it is important to integrate more target-site data into PK/PD models because target-site quantification is not always feasible.^83^ In addition, further fundamental research on protein binding in tissues is needed to improve the estimation of free drug concentrations in tissues. Such studies will also contribute to the refinement of PK/PD models.

## Supplementary Material
