# Supplementary material for: Quantification of Antibiotic Concentration at the Site of Infection: Analytical Challenges and Current Practices
Source: Ther Drug Monit. 2026 Aug 18;48(6):723–30. doi: 10.1097/FTD.0000000000001501 (PMC13747290; doi:10.1097/FTD.0000000000001501)
Supplement: Supplementary file 1 [file tdm-48-723-s001.docx]

antibiotics infection site

670

('antibiotic agent'/exp/mj OR 'antiinfective agent'/mj OR 'antibiotic therapy'/mj OR (antibiotic* OR anti-biotic* OR antimicrob* OR anti-microb* OR antiinfect* OR anti-infect*):ti) AND ('drug tissue level'/mj OR ((infection-site* OR target-site*) NEAR/3 (concentrat* OR distribution* OR quantification*) OR ((level* OR concentration*) NEAR/3 (tissue*) AND (target site* OR infection-site*))):ti) NOT [conference abstract]/lim AND [english]/lim NOT ([animals]/lim NOT [humans]/lim)

987

('antibiotic agent'/exp/mj OR 'antiinfective agent'/mj OR 'antibiotic therapy'/mj OR (antibiotic* OR anti-biotic* OR antimicrob* OR anti-microb* OR antiinfect* OR anti-infect*):ti) AND ('drug tissue level'/de OR ((infection-site* OR target-site*) NEAR/3 (concentrat* OR distribution* OR quantification*) OR ((level* OR concentration*) NEAR/3 (tissue*) AND (target-site* OR infection-site*))):ab,ti,kw) NOT [conference abstract]/lim AND [english]/lim NOT ([animals]/lim NOT [humans]/lim) AND [2000-2025]/py
