# Supplementary material for: Quantification of Antibiotic Concentration at the Site of Infection: Analytical Challenges and Current Practices
Source: Ther Drug Monit. 2026 Aug 18;48(6):723–30. doi: 10.1097/FTD.0000000000001501 (PMC13747290; doi:10.1097/FTD.0000000000001501)
Supplement: Supplementary file 2 [file tdm-48-723-s002.docx]

Table 1: An overview of all challenges including their corresponding references. MD: microdialysis. If no recommendation is mentioned in the paper, a '-' is indicated.

****The described sampling technique does not always correspond to the applied sampling technique, but is related to the described challenge in that paper.***

| **Group of Antibiotics** | **Sampling Technique** | **Matrix** | **Challenges** | **Author’s Recommendations** | **Reference,**  **author, year** |
| --- | --- | --- | --- | --- | --- |
| Antibiotics in general | Tissue biopsy***** | Interstitial fluid | Sample heterogeneity, sampling difficulty | MD | [34]  Brunner, et al., 2005 |
| Antibiotics in general | Tissue biopsy***** | Interstitial fluid | Sampling heterogeneity, sampling difficulty | MD | [8]  Marchand, et al., 2016 |
| Beta-lactams | Tissue biopsy | Synovial tissue, bone | Others | - | [25]  Bahmany, et al., 2024 |
| Beta-lactams | Tissue biopsy | Adipose tissue | Sampling difficulty, unavailability of free drug concentrations | - | [35]  Brunetti, et al., 2016 |
| Beta-lactams | Tissue biopsy | Subcutaneous and fatty tissues | Sample heterogeneity, unavailability of free drug concentrations | MD | [36]  Cinotti, et al., 2018 |
| Beta-lactams | Tissue biopsy | Tissue and interstitial fluid | Sample heterogeneity | - | [37]  Craig, 2000 |
| Beta-lactams | Tissue biopsy | Prostatic tissue | Sample heterogeneity | Attempting to prevent blood contamination during prostate sampling process | [38]  Dariane, et al., 2016 |
| Beta-lactams | Tissue biopsy | Adipose tissue | Sample heterogeneity | MD | [39]  Grupper, et al., 2017 |
| Beta-lactams | Tissue biopsy***** | Epithelial lining fluid | Sampling difficulty, others | MD | [40]  Felton, et al., 2014 |
| Beta-lactams | Tissue biopsy | Fat, synovium, bone | Unavailability of free drug concentrations | - | [23]  Montreuil, et al., 2024 |
| Beta-lactams | Tissue biopsy | Prostate tissue | Sample heterogeneity | - | [41]  Onita, et al., 2025 |
| Beta-lactams | Tissue biopsy | Lung tissue | Sampling heterogeneity, sampling difficulty | MD | [15]  Paal, et al., 2021 |
| Beta-lactams | Tissue biopsy | Soft tissue and bone | Sample heterogeneity, unavailability of free drug concentrations | - | [42]  Sanders, et al., 2019 |
| Beta-lactams | Tissue biopsy | Fat tissue | Sample heterogeneity | - | [43]  Takayama, et al., 2022 |
| Beta-lactams | Tissue biopsy***** | Interstitial fluid | Unavailability of free drug concentrations | MD | [44]  Tomaselli, et al., 2003 |
| Beta-lactams | Tissue biopsy***** | Interstitial fluid | Sampling difficulty | Measuring interstitial fluid instead of the tissue | [45]  Varghese, et al., 2014 |
| Beta-lactams | Tissue biopsy***** | Interstitial fluid | Sampling difficulty | MD | [46]  Varghese, et al., 2015 |
| Beta-lactams | Tissue biopsy*****, body fluids***** | Interstitial fluid | Sample heterogeneity, unavailability of free drug concentrations | MD | [47]  Tomaselli, et al., 2004 |
| Beta-lactams | Tissue biopsy*****, sputum***** | Interstitial fluid | Sample heterogeneity, sampling difficulty | Bronchopulmonary sampling + MD | [48]  Herkner, et al., 2002 |
| Beta-lactams, others | Tissue biopsy | Bone, fat, muscle, hematoma | Sample heterogeneity | - | [49]  Lovering, et al., 2002 |
| Beta-lactams | Tissue biopsy | Prostate tissue | Sample heterogeneity | - | [50]  Nishikawa, et al., 2013 |
| Beta-lactams | Tissue biopsy | Tissues in general | Sampling heterogeneity, sampling difficulty | MD | [13]  Roberts and Lipman, 2009 |
| Fluoroquinolones | Tissue biopsy | Bile, soft tissue, bone | Sample heterogeneity | Applying the hexacyanoferrat method (Roche) to correct for blood contamination | [17]  Böttcher, et al., 2001 |
| Fluoroquinolones | Tissue biopsy | Various types | Sample heterogeneity | - | [51]  Edmiston, et al., 2007 |
| Fluoroquinolones | Tissue biopsy***** | Interstitial fluid | Sample heterogeneity | MD | [52]  Joukhadar, et al., 2001 |
| Fluoroquinolones | Tissue biopsy | Liver tissue | Sample heterogeneity | - | [53]  Justinger, et al., 2012 |
| Fluoroquinolones | Tissue biopsy***** | Epithelial lining fluid | Sampling difficulty | Bronchoscopic microsampling | [54]  Yamazaki, et al., 2003 |
| Fluoroquinolones | Tissue biopsy***** | Extracellular fluid | Sample heterogeneity, sampling difficulty, unavailability of free drug concentrations | MD | [55]  Heinrichs, et al., 2018 |
| Fluoroquinolones | Tissue biopsy | Foot tissue | Sampling heterogeneity, sampling difficulty | - | [56]  Majcher-Peszynska, et al., 2011 |
| Fluoroquinolones | Tissue biopsy | Gallbladder tissue | Unavailability of free drug concentrations | - | [57]  Ober, et al., 2009 |
| Fluoroquinolones | Tissue biopsy | Lung tissue, bone | Sampling heterogeneity, sampling difficulty | - | [58]  Krishna, et al., 2007 |
| Fluoroquinolones | Tissue biopsy | Bone | Sample heterogeneity, unavailability of free drug concentrations, others | Using analytical techniques to determine unbound drug concentrations in bone | [59]  Landersdorfer, et al., 2009 |
| Fluoroquinolones | Tissue biopsy***** | Extracellular space fluid | Sampling heterogeneity, sampling difficulty | MD combined with positron emission tomography | [20]  Langer, et al., 2005 |
| Fluoroquinolones | Tissue biopsy | Subcutaneous fat, skull bone, dura mater, brain tissue | Sampling difficulty | MD | [60]  Leone, et al., 2002 |
| Glycopeptides | Tissue biopsy | Bone and articular tissue | Sampling difficulty | - | [61]  Dunne, et al., 2015 |
| Glycopeptides, others | Tissue biopsy | Epithelial lining fluid, soft tissue, lung tissue | Sampling heterogeneity, sampling difficulty | Correction for blood contamination by measuring the hemoglobin concentration | [16]  Stein and Wells, 2010 |
| Macrolides | Tissue biopsy | Tonsillar tissue | Sample heterogeneity | Bacteria commonly involved in pharyngitis/tonsillitis are located in the extracellular fluids, and therefore the efficacy of antibiotics against these micro-organisms is usually predicted by relating plasma concentrations to MIC values | [62]  Baschiera, et al., 2002 |
| Macrolides | Tissue biopsy | Sinus mucosal tissue | Sample heterogeneity, unavailability of free drug concentrations | - | [63]  Fang, et al., 2009 |
| Macrolides, tetracyclines | Tissue biopsy | Sinonasal tissues | Sampling heterogeneity, sampling difficulty, others | Applying newly developed methods to evaluate the distribution of different compartments | [26]  Siu, et al., 2020 |
| Macrolides | Tissue biopsy | Vaginal tissue | Unavailability of free drug concentrations | MD | [64]  Vodstrcil, et al., 2017 |
| Macrolides | Tissue biopsy***** | Interstitial fluid | Sample heterogeneity | MD | [65]  Zheng, et al., 2014 |
| Macrolides | Tissue biopsy | Rectal tissue | Sample heterogeneity, sampling difficulty, unavailability of free drug concentrations | - | [66]  Kong, et al., 2017 |
| Multiple antibiotics | Tissue biopsy***** | Interstitial fluid | Sample heterogeneity, unavailability of free drug concentrations | MD | [30]  Zeitlinger, et al., 2005 |
| Multiple antibiotics | Tissue biopsy | Various types | Sample heterogeneity | - | [67]  Zhu, et al., 2022 |
| Multiple antibiotics | Tissue biopsy | Various types | Sample heterogeneity, unavailability of free drug concentrations | MD | [68]  Müller, et al., 2004 |
| Multiple antibiotics | Tissue biopsy | Bone | Sample heterogeneity, unavailability of free drug concentrations |  | [21]  Landersdorfer, et al., 2009 |
| Multiple antibiotics | Tissue biopsy | Various types | Sample heterogeneity | MD, Imaging Mass Spectrometry | [1]  Jager, et al., 2019 |
| Others | Tissue biopsy | Lung tissue | Sampling difficulty | - | [69]  Kempker, et al., 2018 |
| Others | Tissue biopsy | Joint tissue | Sample heterogeneity | - | [70]  Menetrey, et al., 2019 |
| Others | Tissue biopsy | Lung tissue | Sampling heterogeneity, sampling difficulty | Clamping the pulmonary artery during the procedure, rinsing the tissues briefly | [71]  Strydom, et al., 2019 |
| Antibiotics in general | Tissue biopsy + MD | Various types | Sampling difficulty | Measuring in skeletal muscle when using lung MD | [72]  Dhanani, et al., 2010 |
| Antibiotics in general | Tissue biopsy + MD | Various types | Sample heterogeneity, sampling difficulty, others | MD combined with positron emission tomography | [19]  Gonzalez, et al., 2013 |
| Antibiotics in general | Tissue biopsy + MD | Various types | Sampling difficulty | - | [73]  van Os and Zeitlinger, 2021 |
| Multiple antibiotics | Tissue biopsy + MD | Various types | Sampling heterogeneity, sampling difficulty, others | Applying more novel analytical quantification techniques such as liquid chromatography coupled by mass spectrometry | [3]  Koch, et al., 2022 |
| Multiple antibiotics | Tissue biopsy + MD | Various types | Sampling heterogeneity, sampling difficulty, others | In vivo imaging techniques + MD | [74]  Liu and Derendorf, 2003 |
| Multiple antibiotics | Tissue biopsy + MD | Various types | Sample heterogeneity | - | [75]  Mouton, et al., 2008 |
| Antibiotics in general | MD | Interstitial fluid | Sampling difficulty, others | The need of sensitive analytical quantification techniques in case of low recoveries | [14]  Dhanani, et al., 2024 |
| Antibiotics in general | MD | Interstitial fluid | Others | Application of MD only for hydrophilic drugs | [8]  Marchand, et al., 2016 |
| Antibiotics in general | Bronchopulmonary sampling | Epithelial lining fluid | Sample heterogeneity, sampling difficulty | - | [76]  Abouelhassan and Nicolau, 2022 |
| Beta-lactams | MD | Subcutaneous and fatty tissues | Sampling difficulty | - | [36]  Cinotti, et al., 2018 |
| Beta-lactams, others | MD | Interstitial fluid | Sampling difficulty | - | [77]  Andreas, et al., 2015 |
| Beta-lactams | MD | Extracellular fluid of cerebrospinal fluid, brain tissue | Sampling difficulty | - | [78]  Hosmann, et al., 2018 |
| Beta-lactams | MD | Interstitial fluid | Sampling difficulty, others | Correction with relative recovery combined with higher flow rates in case of small sample volumes | [28]  Hansen, et al., 2018 |
| Beta-lactams | MD | Epithelial lining fluid | Sampling difficulty | - | [40]  Felton, et al., 2014 |
| Beta-lactams | MD | Interstitial fluid | Sampling difficulty | Development of PK/PD models | [15]  Paal, et al., 2021 |
| Beta-lactams | MD | Interstitial fluid | Others | The need of sensitive analytical quantification techniques in case of small sample volumes | [13]  Roberts and Lipman, 2009 |
| Beta-lactams | MD | Microdialysate | Sampling difficulty | - | [22]  Wulkersdorfer, et al., 2024 |
| Fluoroquinolones | MD | Subcutaneous fat, skull bone, dura mater, brain tissue | Others |  | [60]  Leone, et al., 2002 |
| Glycopeptides | Bronchoscopy | Epithelial lining fluid | Sampling difficulty | - | [79]  Lodise, et al., 2011 |
| Macrolides | Local fluid sampling | Sinus fluid | Sampling difficulty | Using a indwelling sinus catheter | [80]  Ehnhage, et al., 2008 |
| Multiple antibiotics | MD | Interstitial fluid | Others | Ultraslow microdialysis | [27]  De La Peña, et al., 2000 |
| Multiple antibiotics | MD | Various types | Others | - | [1]  Jager, et al., 2019 |
| Multiple antibiotics | MD | Interstitial fluid | Others | MD in combination with other methods to improve the development of PK/PD models for a better description of the different compartments | [30]  Zeitlinger, et al., 2005 |
| Multiple antibiotics | Bronchoalveolar lavage | Epithelial lining fluid | Sample heterogeneity | - | [81]  Kiem, et al., 2008 |
| Others | MD | Skeletal muscle and adipose tissue | Sample heterogeneity | Positron emission tomography combined with MD | [18]  Dehghanyar, et al., 2005 |
| Others | MD | Interstitial fluid | Sample heterogeneity | - | [82]  Frossard, et al., 2000 |
| Others | MD | Interstitial fluid | Others | - | [29]  Sahre, et al., 2012 |
| Others | Bronchoscopy | Epithelial lining fluid, alveolar macrophage | Sampling difficulty | - | [83]  Ong, et al., 2005 |
| Polymyxins | MD | Interstitial fluid | Others | Adding albumin, beta-cyclodextrin, polysorbate, and organic solvents to the perfusion solvents | [24]  Matzneller, et al., 2015 |
